# Conservative Treatment of a Gossypiboma Causing Uterine Wound Dehiscence

**DOI:** 10.1155/2013/578027

**Published:** 2013-09-11

**Authors:** Taner A. Usta, Dogukan Yildirim, Sefik E. Ozyurek, Elif C. Gundogdu

**Affiliations:** Department of Obstetrics and Gynecology, Bagcilar Training and Research Hospital, 34200 Istanbul, Turkey

## Abstract

We present a rare case with gossypiboma following cesarean section which led to uterine wound dehiscence. A 30-year-old woman had been submitted to an emergency cesarean section 4 months previously at another hospital. Clinical and ultrasound findings revealed a large intra-abdominal mass and diffuse peritonitis. At laparotomy, a gossypiboma causing an abscess and uterine wound dehiscence with necrosis of the margins was detected. We performed repetitive wound debridements under broad-spectrum antibiotic cover and eventually resutured the incision. Although hysterectomy has so far been the choice of treatment in the literature once a uterine wound dehiscence had occurred, it was possible in this case to preserve the uterus.

## 1. Introduction

The term “gossypiboma,” which is derived from the Latin gossypium (cotton) and Swahili boma (place of concealment), indicates a mass of cotton that is retained inadvertently in the body following surgery. A surgical sponge is the most common type of retained foreign body [1].

Gossypibomas occurs at a frequency of one per 1000–1500 intra-abdominal operations [2]. The majority of the cases described are provided by gynecological surgery but rarely from cesareans [3]. However, the actual incidence of gossypibomas is difficult to estimate because of underreporting.

It is uncommon that retained surgical sponges following cesarean section lead to uterine wound dehiscence. We present a case with gossypiboma, which led to an abscess and uterine wound dehiscence. 

## 2. Case 

**Figure 1:**
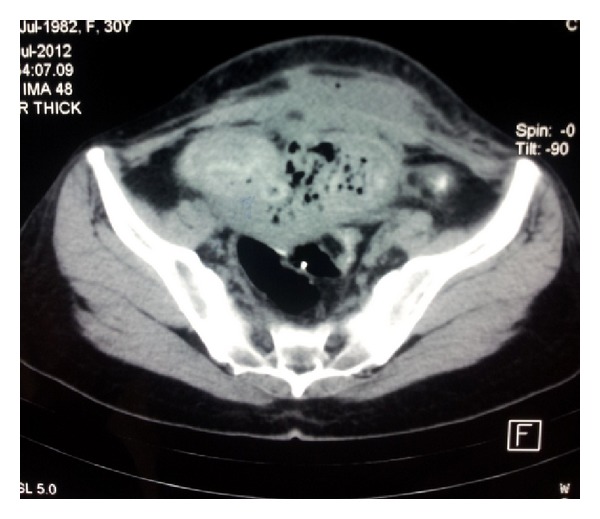
A CT scan showing retained surgical sponge next to the uterus.

**Figure 2:**
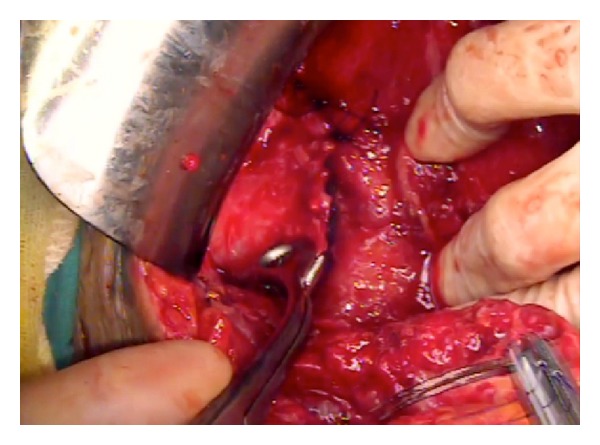
Intraoperative image showing resuturing the incision.

A 30-year-old female patient presented to the emergency room with an unremitting groin pain and vaginal discharge. She underwent an emergency cesarean section at another hospital 4 months earlier. On examination of the patient, hyperemia of the pfannenstiel incision line and a palpable mass right below it was detected. Disseminated tenderness, defence, and rebound were also noted. She was febrile to 38,2°C. On vaginal examination, a purulent leukorrhea and tenderness of the cervix with movement were seen. Transvaginal and transabdominal sonographic examinations were performed, and an approximately 10 cm wide mass lesion image was seen retrovesically. Laboratory investigations revealed increased white cell count (19,400/mm³) and C-reactive protein (214 mg/dL). A direct X-ray graphy and abdominal CT scan of the patient were requested with a preliminary diagnosis of gossypiboma (Figure 1). With the emergence of an acute abdomen picture, the patient was admitted for emergency surgery. An emergency laparotomy was performed. There were adhesions fixating the anterior abdominal to and among the visceral organs. The mass lesion was separated from the surrounding tissues and excised globally. A disseminated infection was observed in the abdomen. With direct observation, it was seen that the cesarean line was completely dehisced and necrosed at the uterocervical level. A swab culture was taken from the margins of the uterine incision which later revealed corynebacterium species. As much debridement as possible was performed around this necrosed uterine incision margins. Due to the extremely disseminated infection and adhesions, a definitive surgical procedure was postponed for a second session. We started administering broad-spectrum antibiotics after surgery. Yet, on the postoperative day 2 following this first intervention, due to the discharge of fecaloid material from the drain, the patient was urgently reopened for an ileal resection and an end-to-end anastomosis of an overlooked bowel injury. Five days following this emergency operation, a final debridement of the uterine incision margins was performed, a foley catheter was placed in the uterine cavity to prevent adhesions and the incision was resutured (Figure 2). The foley catheter was withdrawn 3 days later. She was discharged from hospital on the 7th day following the last operation and is regularly menstruating at present. 

## 3. Discussion

Despite all preventive measures during operation, retained surgical sponges remain a major problem. Risk factors such as emergency surgery, unexpected changes in the surgical procedures, or higher mean body-mass indices were identified for gossypibomas [4]. In our patient, the apparent risk factor was the emergency of the cesarean section.

There are two types of gossypibomas: acute and chronic. The chronic form is characterized by an aseptic fibrinous reaction to the cotton material resulting in development of a mass, with no symptoms or nonspecific subjective symptoms [5]. The acute form is an exudative inflammatory reaction, which leads to the bacterial overgrowth and formation of an abscess. In this scenario, the bowel often bears the brunt of the attempt to expel the foreign body, resulting in intestinal complications like paralytic ileus, perforation, fistula, and intestinal obstructions [6]. The bowel is very vulnerable to peroperative injuries leading to spilling of its content during or following the removal of the foreign body from the inflammatory visceral conglomerate, like in our case. 

Uterine wound dehiscence is a rare, but a serious complication. Reported risk factors for the dehiscence of the lower segment uterine scar following cesarean section are multiparity, infection, and an incision placed too low in the lower uterine segment [7]. Although it is a rare cause of late postpartum hemorrhage, there was no bleeding observed in this case. 

We hypothesized that the gossypiboma played a major role in the intensive cellulitis of the incision leading to the necrosis and separation of the scar. We conducted A MEDLINE search of the literature from 1966 to the present, using the keywords “gossypiboma,” “cesarean delivery,” and “retained surgical sponge,” and did not come up with any study reporting a uterine wound dehiscence accompanying an iatrogenic intraabdominal retention of a foreign material following cesarean section. Only a report was describing a single case of uterine wound dehiscence related to gossypiboma, which was aggressively managed with subtotal hysterectomy and which ended with mortality on the postoperative 4th day [6]. Because of our patient's desire to preserve her future fertility, we performed repetitive wound debridements and eventually resutured the incision under broad-spectrum antibiotic coverage. 

For those patients for whom uterine conservation is not of major importance, hysterectomy is the standard treatment of uterine wound dehiscence [8]. On the other hand, many women are reluctant to have an unexpected hysterectomy [8]. Therefore, conservative approaches including wound debridement and resuturing may be alternatives in selected cases [8, 9]. Nevertheless, this may prove to be very difficult due to the friability of the tissue margins [7].

Although our patient was successfully managed conservatively, consequences for a future pregnancy are unknown. These patients should be informed of the risk of uterine rupture during subsequent pregnancies.
